# Connexin30.2: *In Vitro* Interaction with Connexin36 in HeLa Cells and Expression in AII Amacrine Cells and Intrinsically Photosensitive Ganglion Cells in the Mouse Retina

**DOI:** 10.3389/fnmol.2016.00036

**Published:** 2016-05-27

**Authors:** Arndt Meyer, Stephan Tetenborg, Helena Greb, Jasmin Segelken, Birthe Dorgau, Reto Weiler, Sheriar G. Hormuzdi, Ulrike Janssen-Bienhold, Karin Dedek

**Affiliations:** ^1^Department of Neuroscience and Neurobiology, University of OldenburgOldenburg, Germany; ^2^Research Center Neurosensory Science, University of OldenburgOldenburg, Germany; ^3^Division of Neuroscience, University of DundeeDundee, UK

**Keywords:** connexin, gap junction, primary rod pathway, amacrine cell, melanopsin, ganglion cell, electrical synapse, ipRGC

## Abstract

Electrical coupling via gap junctions is an abundant phenomenon in the mammalian retina and occurs in all major cell types. Gap junction channels are assembled from different connexin subunits, and the connexin composition of the channel confers specific properties to the electrical synapse. In the mouse retina, gap junctions were demonstrated between intrinsically photosensitive ganglion cells and displaced amacrine cells but the underlying connexin remained undetermined. In the primary rod pathway, gap junctions play a crucial role, coupling AII amacrine cells among each other and to ON cone bipolar cells. Although it has long been known that connexin36 and connexin45 are necessary for the proper functioning of this most sensitive rod pathway, differences between homocellular AII/AII gap junctions and AII/ON bipolar cell gap junctions suggested the presence of an additional connexin in AII amacrine cells. Here, we used a connexin30.2-lacZ mouse line to study the expression of connexin30.2 in the retina. We show that connexin30.2 is expressed in intrinsically photosensitive ganglion cells and AII amacrine cells. Moreover, we tested whether connexin30.2 and connexin36—both expressed in AII amacrine cells—are able to interact with each other and are deposited in the same gap junctional plaques. Using newly generated anti-connexin30.2 antibodies, we show in HeLa cells that both connexins are indeed able to interact and may form heteromeric channels: both connexins were co-immunoprecipitated from transiently transfected HeLa cells and connexin30.2 gap junction plaques became significantly larger when co-expressed with connexin36. These data suggest that connexin36 is able to form heteromeric gap junctions with another connexin. We hypothesize that co-expression of connexin30.2 and connexin36 may endow AII amacrine cells with the means to differentially regulate its electrical coupling to different synaptic partners.

## Introduction

In the central nervous system, electrical synapses (gap junctions) constitute a fast means of intercellular communication, directly transmitting electrical signals from one adjacent cell to another. Gap junction channels consist of two hemichannels (connexons), each of which is provided by one cell and formed by a hexamer of connexin (Cx) subunits (Kumar and Gilula, 1996). In the mouse genome, 20 connexin isoforms have been identified (Söhl and Willecke, 2003). Some of these isoforms are able to associate to form heteromeric connexons or heterotypic gap junction channels, with hemi-channel composition differing between the two adjacent cells. As the connexin composition determines the permeability and gating properties of a gap junction channel, a great variety in physiological properties is achieved, which is further enhanced by posttranslational modifications, such as phosphorylation (Söhl and Willecke, 2003).

Gap junctions are found throughout the entire central nervous system but in the retina they are particularly abundant because all major cell types in the retina form coupled networks (Vaney, 1991). In the mouse retina, five different connexin isoforms have been identified in neurons: (1) Cx36, expressed in cone photoreceptors (Feigenspan et al., 2004; Bolte et al., 2015), bipolars (Deans et al., 2002; Han and Massey, 2005), amacrine cells (Feigenspan et al., 2001; Brüggen et al., 2015) and many types of ganglion cells (Pan et al., 2010); (2) Cx45, expressed in bipolar (Maxeiner et al., 2005; Dedek et al., 2006; Hilgen et al., 2011), amacrine (Dedek et al., 2009) and bistratified ganglion cells (Schubert et al., 2005); (3,4) Cx50 and Cx57, both expressed in horizontal cells (Hombach et al., 2004; Dorgau et al., 2015); and (5) Cx30.2, expressed in six types of ganglion cells and an as yet uncharacterized amacrine cell type (Müller et al., 2010a).

In the retina, gap junctions were shown to improve signal-to-noise (DeVries et al., 2002), synchronize firing in ganglion cells (Völgyi et al., 2013) and relay signals into different pathways. Recently, electrical recordings in the rat retina revealed that the melanopsin-expressing, intrinsically photosensitive retinal ganglion cells (ipRGC) are coupled to spiking ON amacrine cells, allowing ipRGCs to propagate their tonic photoresponses to amacrine cells via gap junctions (Reifler et al., 2015). The connexins underlying these electrical synapses in ipRGCs have not yet been identified, but Cx30.2 was suggested to account for the tracer coupling between ipRGCs and GABAergic wide-field amacrine cells (Müller et al., 2010b).

Gap junctions are also critical elements of the primary rod pathway in which rod signals are relayed onto cone pathways by AII amacrine cells. AII cells form gap junctions with two synaptic partners (Hartveit and Veruki, 2012): homocellular gap junctions with other AII cells and heterocellular gap junctions with ON cone bipolar cells. While it has long been known that both types of gap junctions crucially depend on Cx36 (Güldenagel et al., 2001; Deans et al., 2002), some findings suggest the presence of another connexin in AII amacrine cells: homo- and heterocellular gap junctions are differentially modulated (Mills and Massey, 1995) and assembled (Meyer et al., 2014), and appear different in electron micrographs (Anderson et al., 2011). Despite these observations, previous studies implicated Cx36 in the establishment of homomeric connexons only (Teubner et al., 2000; Li et al., 2008) although some studies have suggested the co-expression of Cx45 in AII amacrine cells (Maxeiner et al., 2005; Li et al., 2008). However, Cx36 and Cx30.2 were shown to be co-expressed in inhibitory interneurons in the hippocampus (Kreuzberg et al., 2008), we therefore speculated that Cx30.2 and Cx36 may interact and co-assemble in AII amacrine cells. Therefore, we used a Cx30.2*lacZ* mouse line (Kreuzberg et al., 2006) to extend our studies on Cx30.2 expression in the mouse retina.

We show that Cx30.2 is expressed in ipRGCs and AII amacrine cells of the mouse retina. Moreover, we reveal interaction of Cx36 and Cx30.2 in transfected HeLa cells suggesting that Cx36 is able to form heteromeric gap junctions with another connexin. We propose that this may provide the basis for the differential regulation of Cx36-containing heterocellular and homocellular gap junctions in AII amacrine cells.

## Materials and Methods

Unless mentioned otherwise, reagents and chemicals were purchased from Roth (Karlsruhe, Germany).

### Constructs and HeLa Cell Transfections

Full-length Cx30.2 and Cx36 constructs (mouse sequences), untagged or tagged with enhanced green-fluorescent protein (EGFP), were cloned in pRK5 (BD Pharmingen, San Diego, CA, USA; Helbig et al., 2010). All constructs were sequenced for accuracy. HeLa cells were transiently transfected with the calcium-phosphate precipitation method. Briefly, 24 h before transfection, HeLa cells were plated at a density of 1 × 10^5^ in a 6 cm diameter dish including two coverslips, in 5 ml Dulbecco’s Modified Eagle Medium (Biochrom GmbH, Berlin, Germany), supplemented with 10% fetal bovine serum (Biochrom). For transfection, precipitation solution, including 25 μg/ml DNA, was applied 48 h before cell lysis. For co-expression of connexin constructs, cells were transfected with a plasmid mixture containing equal amounts of both constructs.

### Reverse Transcription Polymerase Chain reaction (RT-PCR)

Retinal total RNA was extracted using the TriFast^TM^ reagent (PeqLab, Erlangen, Germany) according to the manufacturer’s instructions. Residual genomic DNA contamination was eliminated by treatment with DNaseI (Amplification Grade; Invitrogen, Darmstadt, Germany). The first-strand cDNA synthesis was carried out using 1 μg of total RNA, 1× first-strand buffer (Invitrogen), Oligo(dT)15 primer (20 ng/μl; Promega, Mannheim, Germany), dNTPs (0.4 mM each; Carl Roth, Karlsruhe, Germany), RiboLock RNase Inhibitor (1.6 U/μl; Thermo Fisher Scientific, Schwerte, Germany) and SuperScript III reverse transcriptase (8 U/μl) according to the manufacturer’s manual. Forty nanogram of the transcribed cDNA were subsequently used as PCR template in reaction buffer (Qiagen, Hilden, Germany) containing MgCl_2_ (1.5 mM), 0.2 mM dNTPs (Carl Roth), 0.4 μM primer and HotStar Taq polymerase (0.5 U/μl; Qiagen). The quality of the cDNA was tested using intron-spanning primers for β-actin (usp: 5′-tgttaccaactgggacgaca-3′; dsp: 5′-aaggaaggctggaaaagagc-3′; product size: 573 bp for cDNA and 1027 bp for gDNA). To amplify partial Cx30.2 cDNA, a specific primer set (usp: 5′-atgcaccaggccagcaaggag-3′; dsp: 5′-ccgcgctgcgatggcaaagag-3′; product size: 422 bp) and 1× Q-solution (Qiagen) was used.

### Generation of Anti-Connexin30.2 Antibodies

Cx30.2 antibodies were raised in rabbit and guinea pig (Pineda Antibody Service, Berlin, Germany). The peptides used for immunization comprised the last 20 amino acids of the C-terminal end of mouse Cx30.2 (rabbit antibodies) or amino acids 92–109 of mouse Cx30.2, which form part of the cytoplasmic loop (guinea pig antibodies). Antibodies were affinity-purified using the immunization peptides.

### Immunoprecipitation and Western Blot Analysis

Immunoprecipitation (IP) experiments were performed using the μMACS™ GFP Isolation Kit (Miltenyi Biotec GmbH, Bergisch Gladbach, Germany) following the manufacturer’s instructions. HeLa cells were harvested 48 h after transfection and homogenized in 350 μl IP buffer, containing 0.5% NP-40, 20 mM Tris, 60 mM NaCl (pH 7.4), and phosphatase and protease inhibitors (Roche Diagnostics, Mannheim, Germany). Homogenates were incubated for 1 h on ice and centrifuged for 10 min at 10,000 g at 4°C. The supernatant was removed and incubated for 30 min with 20 μl of magnetic beads which were covalently coupled to an anti-GFP antibody (Table 1). After several washes, adsorbed proteins were eluted with pre-heated (95°C) elution buffer, containing 50 mM Tris HCl (pH 6.8), 50 mM DTT, 1% SDS, 1 mM EDTA, 0.005% bromophenol blue, 10% glycerol. SDS-PAGE (10% gels) and Western blot analysis were performed with solubilized (extracted) membrane proteins and precipitates prepared from HeLa cell homogenates. Enhanced chemiluminescence-mediated immunodetection was carried out following a standard protocol. Nitrocellulose membranes were incubated with primary antibodies (Table 1) diluted in TBS-Tween (20 mM Tris/HCl, 150 mM NaCl, 0.2% Tween, pH 7.4) overnight at 4°C. Immunoreactive proteins were visualized using horseradish peroxidase-conjugated secondary antibodies (1:2000–1:3000, diluted in TBS-Tween with 2% powdered milk; BioRad, Munich, Germany) and the Enhanced Chemiluminescence Detection kit (Pierce, Rockford, IL, USA), following the manufacturer’s instructions.

**Table 1:** **List of primary antibodies used in this study**.

Antibody	Antigen	Host, type	Dilution	Source (Cat. No.)
Calbindin D-28K	Recombinant rat calbindin D-28K	Rabbit, polyclonal	1:500	Swant, Marly, Switzerland (CB-38)
Calretinin	Guinea pig calretinin, full length amino acid sequence	Goat, polyclonal	1:500	Merck-Millipore, Darmstadt, Germany (AB 1550)
ChAT	Human placental choline acetyltransferase	Goat, polyclonal	1:250	Merck-Millipore (AB 144P)
Cx30.2	Peptide derived from amino acids 92–109 of mouse Cx30.2	Guinea pig, polyclonal	1:500	Pineda Antibody Service, Berlin, Germany
Cx30.2	Peptide derived from the last 20 amino acids of the C-terminal end of mouse Cx30.2	Rabbit, polyclonal	1:5000 (wb) 1:500 (He)	Pineda Antibody Service
Cx36	C-terminal region of rat and mouse Cx36, derived from amino acids 286–303	Mouse, monoclonal	1:500	Invitrogen (37-4600)
Disabled-1	GST-mDisabled-1 fusion protein, corresponding to amino acids 107–243	Rabbit, polyclonal	1:500	Gift from Dr B. Howell, NIH
β-Galactosidase	Full length purified β-gal from *Escherichia coli*	Chicken, polyclonal	1:500	Abcam, Cambridge, MA, USA (ab9361)
GFP	Full-length *Aequorea victoria* GFP	Mouse, monoclonal	1:5000	Clontech, Mountain View, CA, USA (632380)
Melanopsin	15 most N-terminal amino acids of the mouse melanopsin extracellular domain	Rabbit, polyclonal	1:5000	Advanced Targeting Systems, San Diego, CA, USA (AB-N38, UF006)
Parvalbumin	Rat muscle parvalbumin	Rabbit, polyclonal	1:5000	Swant (PV28)

*Dilutions were identical for labeling of cryosections, retinal whole-mounts, Western blot (wb) and HeLa cell experiments (He) if not stated otherwise*.

### Animals and Tissue Preparation

All procedures were approved by the local animal care committee (*Niedersaechsisches Landesamt fuer Verbraucherschutz und Lebensmittelsicherheit*) and were in compliance with the guidelines for the welfare of experimental animals issued by the European Communities Council Directive of 24 November 1986 (86/609/EEC) and the laws of the Federal Government of Germany (*Tierschutzgesetz*; BGBl. I S. 1206, 1313 and BGBl. I S. 1934).

To generate the Cx30.2^lacZ^ mouse line, the coding region of *Cx30.2* was deleted and replaced with *lacZ* reporter DNA, containing a nuclear localization signal, under the control of the endogenous *Cx30.2* promoter (Kreuzberg et al., 2006). Cx36-deficient mice were generated as described (Deans et al., 2002).

Adult mice (aged 2–12 months) were euthanized with CO_2_ followed by cervical dislocation. Eyes were enucleated, and cornea, lens and vitreous body were removed in 0.1 M phosphate buffer (PB, pH 7.4) for immunohistochemical experiments. For tracer-coupling experiments, injections were carried out in Ringer’s solution (in mM: 110 NaCl, 2.5 KCl, 1 CaCl_2_, 1.6 MgCl_2_, 10 D-glucose, 22 NaHCO_3_) at room temperature, aerated with 95% O_2_ and 5% CO_2_ and resulting in an pH of 7.4 when reaching the bath chamber. For dye injections in vibratome slices, the pH of the Ringer’s solution was adjusted to 7.4 using 5 mM HEPES instead of NaHCO_3_.

### Intracellular Dye and Tracer Injections

For the dye injections of Cx30.2^lacZ^-positive cells, retinas were isolated from the eyecup, bisected and embedded in 2% agar-agar in Ringer’s solution. Vertical vibratome sections of 200 μm were prepared as described previously (Dedek et al., 2006). The slices were stained with fluorescein-di-beta-D-galactopyranoside (FDG; Sigma), a fluorogenic substrate for β-galactosidase (β-gal) which can be used to visualize *lacZ*-expressing cells. FDG was applied in Ringer’s solution by adding 1.5 μl of a 120 mg/ml stock solution in dimethyl sulfoxide (Sigma) to the bath chamber and washing it out with the perfusion after 1–2 min. Labeled cells were injected under visual control with sharp microelectrodes (120–180 MΩ) filled with 5 mM Alexa Fluor 488 potassium hydrazide (Invitrogen, Karlsruhe, Germany) diluted in 0.2 M KCl, pH 7.4. Slices were then fixed in 4% paraformaldehyde (PFA) and processed for microscopy.

For tracer-coupling experiments, isolated retinae were cut into halves or quarters and mounted, ganglion-cell side up, on black nitrocellulose filters (Milipore, Billerica, MA, USA). AII amacrine cells were injected either “blindly” by advancing the electrode to the proximal inner nuclear layer (INL) and impaling random cell somata, or under visual control after incubation in 0.1 mM DAPI diluted in Ringer’s solution for 20–60 min to visualize the nuclei of amacrine cells in the proximal INL. Microelectrodes (120–180 MΩ) were filled with 5 mM Alexa Fluor 488 and 4% (w/v) neurobiotin (Vector Laboratories, Burlingame, CA, USA). The Alexa dye was iontophoresed with 0.5 nA square pulses of 500 ms at 1 Hz for 1–2 min to visualize the cell’s morphology; when an AII cell was successfully impaled, the current was reversed to inject the positively charged neurobiotin (6–7 min). After the injection, the tracer was allowed to diffuse for at least 15 min before the specimen was fixed in 4% PFA for 10–20 min. We never observed a systematic increase in AII cell tracer coupling with longer diffusion times. The neurobiotin was visualized with DyLight549-conjugated streptavidin (Jackson ImmunoResearch, West Grove, PA, USA).

### Immunostaining of Frozen Sections, Whole-Mount Retinas and HeLa Cells

Eyecups were fixed in 4% PFA for 10 min, washed, cryoprotected with 30% sucrose in 0.1 M phosphate buffer (PB, pH 7.4) overnight and embedded in TissueTek as described (Dedek et al., 2009). Cryosections of 20 μm were cut, dried and blocked with 5% ChemiBLOCKER (Millipore, Billerica, MA, USA), 10% normal goat serum or donkey serum in PB. Incubation with primary antibodies (Table 1) was carried out at 4°C overnight and, after extensive washing, with secondary antibodies for 2 h at room temperature.

Whole-mount retinae were mounted on black filter paper, fixed in 4% PFA for 10 min, washed and blocked with 10% normal goat serum or donkey serum in PB. Primary antibodies were incubated for 3–7 days at 4°C, and secondary antibodies for 2–5 days at 4°C. For melanopsin immunolabeling, whole-mounts were fixed overnight and incubations were carried out in 50 mM TRIS buffer (pH 7.5) containing 1.5% NaCl, instead of PB.

For immunocytochemistry, 48 h after transfection, HeLa cells were fixed on coverslips with 2% PFA for 15 min. The subsequent steps for immunolabeling were the same as for cryosections.

In all blocking and antibody incubation steps, the solutions contained 0.3–0.5% Triton X-100 (and 0.05% NaN_3_ for cryosections and retinal whole-mounts). Secondary antibodies were conjugated to Alexa 488, Alexa 568 or Alexa 594 (1:500, Invitrogen). For controls, the primary antibody was omitted; occasional labeling of blood vessels was observed for secondary anti-mouse antibodies. After final washes, all specimens were mounted in Vectashield.

### *LacZ* Staining in Retinal Cryosections

Vertical cryosections (20 μm) from Cx30.2^lacZ/lacZ^ mice were prepared as above and stained for β-gal activity as described previously (Kranz et al., 2013). Briefly, slices were washed in *lacZ* washing solution and incubated with the β-gal substrate X-gal for 1–7 days at 37°C.

### Electroretinogram Recording

Scotopic electroretinograms (ERGs) were recorded as described previously (Kranz et al., 2013) from 10 mice of each genotype. Full-field stimulation comprised 10 light intensities, ranging from −3.5 to 1 log cds/m^2^. Data analysis was performed using Chart v5.5 (AD Instruments, Hastings, UK). Data was evaluated for statistical differences using two-way analysis of variance (ANOVA) in Prism 5 (GraphPad Software, La Jolla, CA, USA).

### Image Acquisition and Analysis

**Figure 1:**
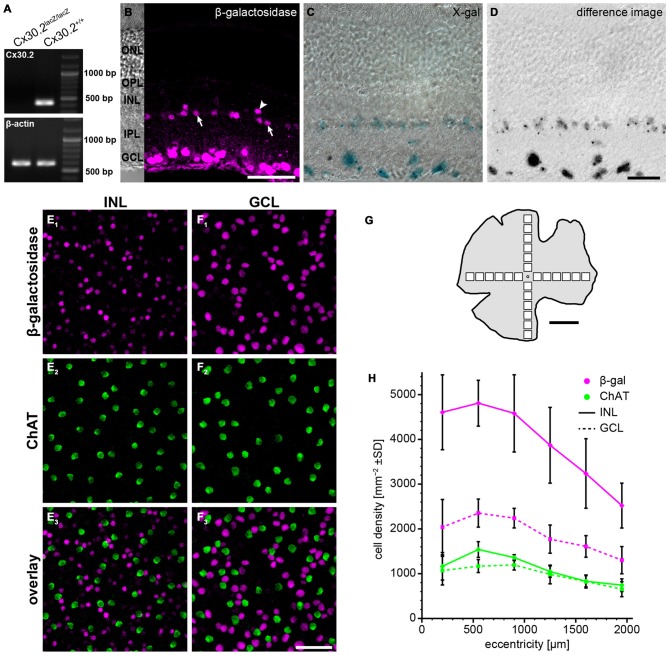
**Reporter gene expression in the Cx30.2^lacZ/lacZ^ retina is confined to the ganglion cell layer (GCL) and the proximal inner nuclear layer (INL). (A)** Successful deletion of Cx30.2 was confirmed by RT-PCR. The Cx30.2-specific transcript was only detected in Cx30.2^+/+^ and not in Cx30.2^lacZ/lacZ^ mice. Intron-spanning actin primers were used to prove that samples were not contaminated by genomic DNA: only the 573 bp for cDNA is visible whereas the 1027 bp for genomic DNA was not detected. **(B)** Immunofluorescence staining for β-galactosidase (β-gal) in a vertical section of the Cx30.2^lacZ/lacZ^ retina. The inset on the left is a transmission image of the same area indicating the layers of the retina (ONL, outer nuclear layer; OPL, outer plexiform layer; INL, inner nuclear layer; IPL, inner plexiform layer; GCL, ganglion cell layer). In the INL, immunoreactive cells are visible right at the border between INL and IPL (arrows) or a bit more distal (arrowhead). **(C)** X-gal staining of a Cx30.2^lacZ/lacZ^ retina. Blue signals indicate β-gal expression. **(D)** Same image as in **(C)** showing the extracted signals of the X-gal staining along a Z-series and confirming the exclusive expression in the GCL and the proximal INL. **(E,F)** Whole-mount stainings for β-gal (E_1_,F_1_) and choline acetyltransferase (ChAT, E_2_,F_2_) in the INL **(E)** and GCL **(F)** to quantify β-gal expression. ChAT signals never colocalized with β-gal signals. **(G)** Schematic of a whole-mount retina indicating the approximate locations for quantification. **(H)** Quantification of β-gal expressing cells depending on the eccentricity within the retina (mean ± SD, *n* = 8 central-to-peripheral axes). Scale bars: **(B,E,F)** 50 μm; **(C,D)** 20 μm; **(G)** 1 mm.

*LacZ*-stainings with X-gal (Figure 1C) were imaged on a Zeiss Axiophot 2 equipped with a 63× plan Apochromat oil immersion objective (NA 1.4) and a Zeiss Axiocam MRc. *Z*-series were taken at manually chosen focal planes. In order to detect even very faint X-gal signals and to distinguish them from small debris particles, the blue X-gal signals (which maximally absorb in the red spectrum of visible light) were extracted in ImageJ (NIH, Bethesda, MD, USA) by generating minimum projections of the image stacks, splitting the RGB images into separate channels and generating difference images of the red and the blue channel (Figure 1D).

Intracellular dye injections into FDG-labeled cells in vibratome slices (Figures 3A,B) were documented during the injections on a Zeiss Axioskop equipped with a 40 × Achroplan water immersion objective (NA 0.75) and a Leica DFC320 camera. These RGB photomicrographs were converted to 8-bit, recolored and aligned with confocal images using ImageJ.

Confocal images of retinal sections and whole-mounts were taken on Leica SP2 or SP8 TCS SL microscopes with a 40× HCX PL APO oil immersion objective (NA 1.25 or 1.3). For HeLa cells, a 63× HCX PL APO oil immersion objective (NA 1.32) was used.

**Figure 2:**
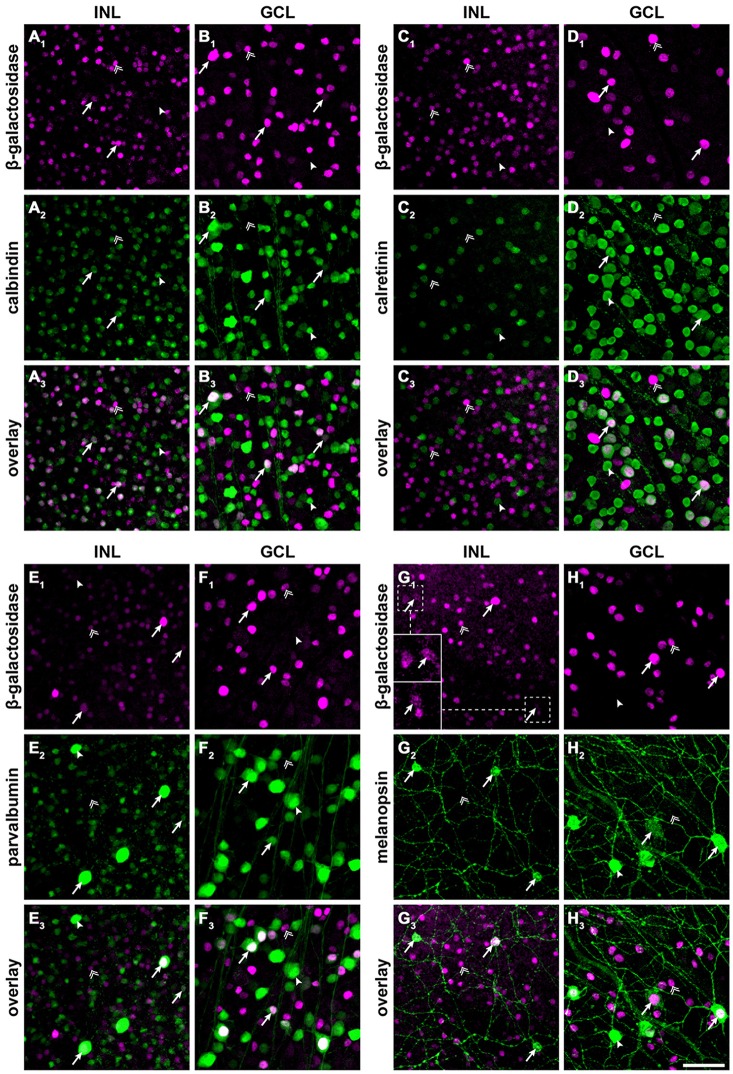
**Cx30.2^lacZ^-expressing neurons belong to several different populations of amacrine and ganglion cells, including intrinsically photosensitive retinal ganglion cells (ipRGCs). (A–H)** Double immunofluorescence stainings for β-gal **(A_1_–H_1_)** and calbindin **(A_2_,B_2_)**, calretinin **(C_2_,D_2_)**, parvalbumin **(E_2_,F_2_)**, and melanopsin **(G_2_,H_2_)** in the INL **(A/C/E/G)** and GCL **(B,D,F,H)** of Cx30.2^lacZ/lacZ^ whole-mount retinae. **(A_3_–H_3_)** show overlays of the respective single channels. Arrows indicate example cells that express both antigens, double arrows indicate cells that express β-gal but lack the respective marker, and arrowheads indicate cells expressing the marker but lacking β-gal. Insets in **(G_1_)** show intensity-enhanced magnifications of the areas marked by dashed squares to verify colocalization with melanopsin. Scale bar: 50 μm.

For the quantification of β-gal-positive cells (Figures 1G,H), confocal stacks comprising the ganglion cell layer (GCL), inner plexiform layer (IPL) and INL were taken along the dorso-ventral and naso-temporal axes of the retina at intervals of 350 μm. At each location, a square area of 200 × 200 μm was evaluated. Cells were counted manually in maximum projections of partial confocal stacks using the cell counter plugin of ImageJ. If in doubt, the original stacks were re-examined. As proposed by Jeon et al. (1998), choline acetyltransferase (ChAT)-immunoreactive cells were also counted and used as a standard. Our average numbers of ChAT-positive cells deviate from the published numbers by less than 4% (INL: −1.3%, GCL: −3.7%). Since we did not observe obvious differences between the major axes of the retina, all data are presented as mean ± standard deviation of the mean (SD) as a function of eccentricity. Colocalization of β-gal-immunoreactivity with different calcium-binding proteins (CaBPs, Figure 2, Table 2) was evaluated in a similar way in randomly chosen mid-peripheral locations (*n* = 2–4 scans from 2 to 4 retinae). For the density of Cx36-immunoreactive clusters in wild-type and Cx30.2^lacZ/lacZ^ retinae (Figure 6J), confocal stacks were taken in four retina pieces obtained from three animals per genotype, deconvolved with a theoretical point spread function using Huygens Essential deconvolution software (SVI, Hilversum, Netherlands) and processed afterwards with Fiji (http://fiji.sc/Fiji; Schindelin et al., 2012). Sixty-four regions of interest (four per image; 22,900 μm^2^ in total per genotype and sublayer) were positioned randomly in the middle of the ON- and OFF-sublayers of the IPL and then shifted to the nearest place that did not contain any blood vessels, if necessary. After segmentation (*Li*’s threshold) and separation of fused particles using the watershed algorithm, clusters were counted automatically.

**Table 2:** **Colocalization (coloc.) of β-galactosidase immunoreactivity with different calcium-binding proteins (CaBP)**.

	(*n*)	CaBP-positive cells	β-gal-positive cells	coloc.	%-coloc. (β-gal/CaBP)	%-coloc. (CaBP/β-gal)
**INL**
Calretinin	2	99 ± 11	243 ± 69	1	0.5	0.2
Parvalbumin	4	329 ± 52	308 ± 64	59 ± 13	18 ± 3	19 ± 1
Calbindin	4	432 ± 76	293 ± 38	169 ± 33	40 ± 9	57 ± 6
**GCL**
Calretinin	3	191 ± 38	125 ± 39	72 ± 19	38 ± 10	59 ± 11
Parvalbumin	4	149 ± 16	174 ± 24	63 ± 9	42 ± 5	37 ± 6
Calbindin	4	193 ± 55	149 ± 20	44 ± 27	21 ± 7	29 ± 16

*(n) is the number of retinae; a square area of 300 × 300 μm^2^ was evaluated in each retina. All values are given as mean ± standard deviation*.

To quantify the size of gap junctional clusters between adjacent HeLa cells, confocal stacks were deconvolved with a theoretical point spread function using Huygens Essential and processed afterwards with Fiji. Stacks were normalized to the respective stack histogram and segmented automatically (*Otsu*’s threshold). The *3D Simple Segmentation* algorithm was used for the automatic detection of gap junction clusters, which were then measured in volume using the *3D Manager* (*Fiji* plugin). For each combination of plasmids, the number and size of clusters were determined for three independent transfection experiments. Data were evaluated for statistical differences using the Kruskal-Wallis test in Prism, with adjusted *p*-values for multiple comparisons.

All images were adjusted in brightness and contrast for presentation purposes using ImageJ or Fiji. Confocal micrographs are presented as maximum or average projections of the image stacks, except for the rotated projections in Figures 6C,F which are 3D-projections. HeLa cell stainings are represented as maximum projections of three subsequent scans. Some confocal images of nuclear or somatic immunostainings were filtered with a Gaussian (*r* = 2 pixels) prior to projection in order to reduce noise.

## Results

### *LacZ* Expression in Cx30.2-Deficient Mice

To study the expression of Cx30.2 in the mouse retina, we used a mouse line in which the coding region of Cx30.2 was replaced by *lacZ* reporter DNA (Kreuzberg et al., 2006; Müller et al., 2010a), coding for β-gal. Successful deletion of Cx30.2 from the mouse retina was confirmed by RT-PCR (Figure 1A): the Cx30.2-specific band (422 bp) was only detected in wild-type and not in Cx30.2-deficient retina. Contamination by genomic DNA was excluded using intron-spanning actin primers. Labeling retina sections with anti-β-gal antibodies revealed *lacZ* expression exclusively in the proximal INL and the GCL (Figure 1B). *LacZ* staining with the non-fluorogenic substrate X-gal showed precipitates in the same layers (Figures 1C,D), suggesting Cx30.2 expression in amacrine and ganglion cells only. Consistent with an earlier study (Müller et al., 2010a), staining for β-gal and X-gal indicated Cx30.2 expression in at least two different populations of amacrine cells: one population with the soma at the border between INL and inner IPL (Figure 1B, arrows) and a second, with fewer positive cells, more distally (Figure 1B, arrowhead). Likewise, several different populations of neurons were labeled in the GCL as discernible by the different sizes of labeled somata (Figures 1B–D). β-gal expression was similar in heterozygous and homozygous Cx30.2^lacZ^ mice. We never detected *lacZ-expressing* cells in the distal INL (bipolar or horizontal cells) or in photoreceptors. In contrast to an earlier study (Manasson et al., 2013), we did not find any indication for Cx30.2 expression in endothelial cells in the mouse retina: *lacZ-expressing* nuclei never showed the typical elongated shape of endothelial nuclei and were never observed in areas with retinal blood vessels.

To estimate the density of *lacZ*-expressing cells, we labeled whole-mount retinas for ChAT (Figures 1E–H). ChAT is a marker for starburst amacrine cells which form two almost identical populations of amacrine cells, whose cell bodies are located in the INL and GCL. As starburst cell density is well known—they make up 3% of all amacrine cells in the INL and 11.5% of all cells in the GCL (Jeon et al., 1998)—we used them as a reference population. *LacZ*-positive cells in the INL showed a much higher density than starburst amacrines (Figure 1H; ~4800 vs. 1500 cells/mm^2^ at 500 μm eccentricity); we never found *lacZ* expression in ChAT-labeled cells. Using starbursts as a reference, we estimated that 9% of all amacrine cells in the INL express Cx30.2. The relatively high density confirms the presence of several *lacZ-positive* populations in the INL. This supports an expression in narrow-field amacrine cells because these amacrines show high densities to allow full coverage of the retina.

The density of *lacZ*-expressing somata in the GCL was also higher than the number of ChAT-expressing starburst cells, in line with our previous results that several populations of ganglion cells express Cx30.2 (Müller et al., 2010a). Assuming that Cx30.2-expressing cells in the GCL comprise no or very few displaced amacrine cells (Müller et al., 2010a), the average of 1886 cells/mm^2^ in the GCL adds up to about 54% of all ganglion cells.

To further characterize *lacZ*-positive cells, we labeled whole-mount retinas for β-gal and either calbindin (Figures 2A,B), calretinin (Figures 2C,D) or parvalbumin (Figures 2E,F), which are all markers for amacrine and ganglion cells (Haverkamp and Wässle, 2000). In the INL, we did not find colocalizaton of β-gal with calretinin (Figure 2C) but a high degree of overlap with calbindin-positive (Figure 2A) and parvalbumin-positive somata (Figure 2E; please see Table 2 for numbers). The majority of cells positive for β-gal and calbindin or parvalbumin most likely represented amacrine cells (Figures 2A,E) because of their high density and small somata. However, some parvalbumin- and β-gal-positive somata in the INL were very large and most likely represented displaced ganglion cells (Figure 2E). We found two different examples of displaced ganglion cells which expressed either both antigens or only one of the two.

In the GCL, all three markers showed considerable colocalization with β-gal (Figure 2; Table 2). As calretinin and parvalbumin were reported to demonstrate a differential expression among different types of ganglion cells (Kim and Jeon, 2006; Lee et al., 2010), these data again confirm Cx30.2 expression in several populations of ganglion cells.

Taken together, we find Cx30.2 expression restricted to the INL and GCL, in a variety of amacrine and ganglion cell types.

### *LacZ* Expression in Melanopsin-Positive Ganglion Cells

In an earlier study, we identified Cx30.2 expression in six different types of ganglion cells (Müller et al., 2010a), in which Cx30.2 may contribute to homotypic coupling among ganglion cells of the same type or to heterotypic coupling to amacrine cells. Heterotypic coupling has also been demonstrated for intrinsically photosensitive retinal ganglion cells (ipRGCs): three subtypes of ipRGCs (M1–M3) were shown to be tracer-coupled to displaced amacrine cells (Müller et al., 2010b); and recently, Reifler et al. (2015) reported that ipRGCs provide input to displaced, spiking ON amacrine cells via electrical synapses. As the gap junction protein expressed by ipRGCs is not known, we double-labeled Cx30.2^lacZ/lacZ^ retinas for β-gal and melanopsin (Figures 2G,H), the photopigment expressed by ipRGCs. Indeed, we found strong colocalization of both signals in large somata in the GCL (Figure 2H) and in ipRGCs displaced to the INL. However, some melanopsin-positive somata displayed very weak or no β-gal immunoreactivity, suggesting that not all ipRGCs express Cx30.2 (Figure 2H, arrowhead).

In summary, we identified Cx30.2 as a strong candidate for forming the gap junctions between ipRGCs and displaced amacrine cells.

### *LacZ* Expression in AII Amacrine Cells

**Figure 3:**
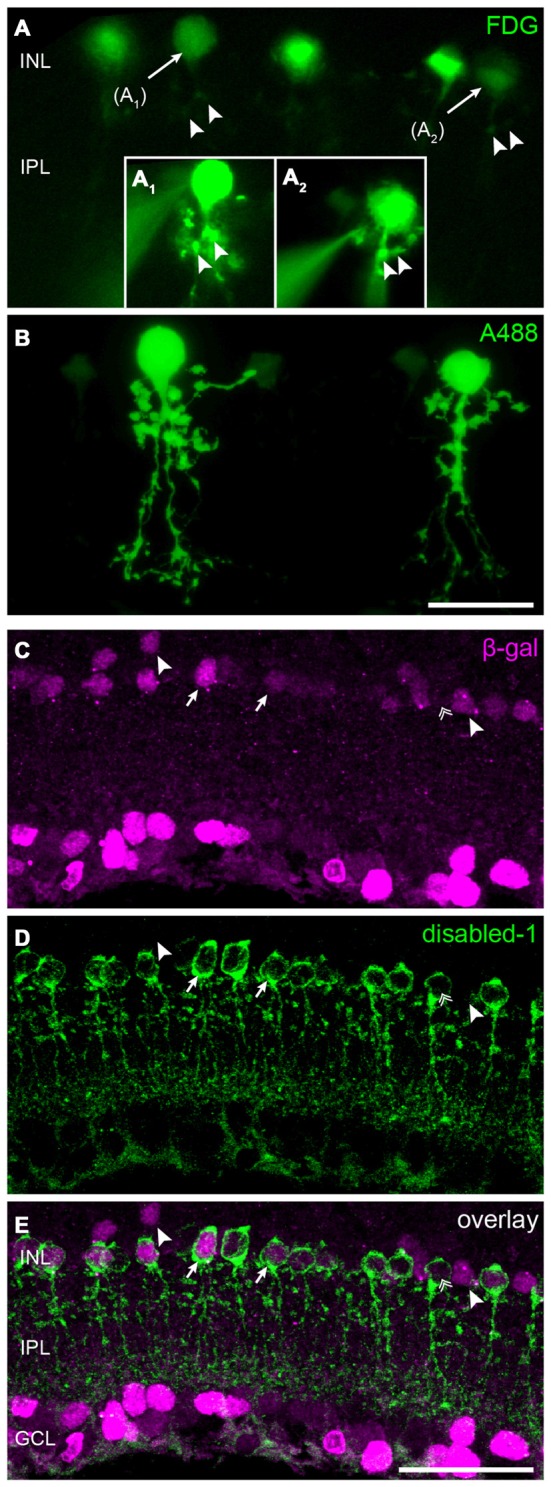
**Cx30.2^lacZ^-expression in AII amacrine cells. (A)** *Ex vivo* labeling of Cx30.2^*lacZ*^-expressing cells in a vibratome slice of a Cx30.2^lacZ/lacZ^ retina using fluorescein-di-β-D-galactopyranoside (FDG). Arrows indicate cells **(A_1_,A_2_)** that were chosen for the intracellular injection of Alexa 488. Insets **(A_1_,A_2_)** document the intracellular injection; the injection electrode is visible on the left side of the images. Arrowheads in **(A)** indicate morphological features of the injected cells that help confirming the successful impalement of the targeted cells. **(B)** Confocal micrograph showing the typical AII morphology of the injected cells from **(A)** with lobular appendages in the distal IPL and arboreal dendrites in the proximal INL. **(C–E)** Double labeling for β-gal **(C)** and the AII marker disabled-1 **(D)** verifies the Cx30.2^*lacZ*^-expression in most AII amacrine cells (**E**, arrows). Some AII cells, however, display very little or no Cx30.2^lacZ^-expression (double arrow); also, few non-AII amacrine cells are positive for β-gal (arrowheads). Scale bars: **(A–D)** 20 μm; **(E)** 50 μm.

The high density of *lacZ*-expressing neurons in the INL suggested Cx30.2 expression in narrow-field amacrine cells of which the mouse retina contains at least 13 different types (Pang et al., 2012; Lee et al., 2015). As specific markers are only available for some of these types, we took a different approach to further characterize Cx30.2-expressing cells in the INL. We used a fluorogenic substrate of β-gal to label *lacZ*-positive cells in retina vibratome sections (Figures 3A,B). Pre-labeled somata were subsequently targeted with an Alexa dye-filled microelectrode (Figure 3A_1,2_) and images were first taken in the living tissue and, after fixation, cell identity was confirmed with confocal microscopy (Figure 3B). These experiments revealed the morphology of *lacZ*-expressing amacrine cells: (1) the cells’ somata were located right at the border between INL and IPL; and (2) cells had a thick primary dendrite which (3) branched into lobular appendages in the OFF sublamina and fine, arboreal processes, crossing the entire ON sublamina of the IPL. This morphology is typical for AII amacrine cells (Hartveit and Veruki, 2012) and was observed for all reliably injected amacrine cells (*n* = 12). To confirm this finding, we labeled retina cryosections for β-gal and disabled-1 (Figures 3C–E), a marker for AII amacrine cells in the mouse retina (Rice and Curran, 2000). Most cell somata labeled for disabled-1 were also positive for β-gal (Figure 3, arrows). However, some AII cells lacked β-gal immunoreactivity (Figures 3C–E, double arrow). Likewise, some β-gal-positive somata between the AII cells or located more distally from the INL/IPL border lacked disabled-1 immunoreactivity (Figures 3C–E, arrowheads).

In summary, these data provide evidence that AII amacrine cells, known to couple via Cx36, additionally express Cx30.2, which is also made by other—yet unidentified—amacrine cell types.

### Characterization of Anti-Cx30.2 Antibodies

**Figure 4:**
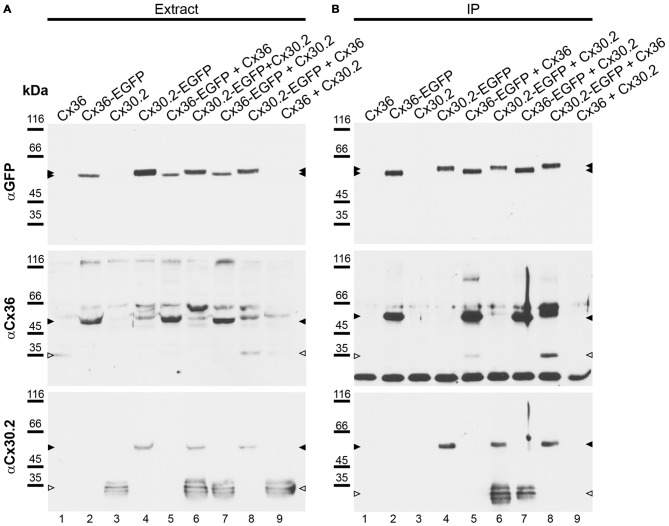
**Newly generated anti-Cx30.2 antibodies (rabbit) are specific and reveal interaction of Cx30.2 and Cx36. (A)** Western blot analysis of HeLa cell extracts transfected with different combinations of Cx30.2 and Cx36 and their enhanced green fluorescent protein (EGFP)-tagged variants. Blots were probed with anti-GFP, anti-Cx36 and newly generated anti-Cx30.2 antibodies from rabbit. Anti-Cx36 and anti-Cx30.2 antibodies recognize the respective connexin (**A,B**, open arrowheads) and its fusion variant (**A,B**, filled arrowheads). Please note that several bands become visible for untagged Cx30.2, pointing to different phosphorylation states (also see Supplementary Figure 1). These bands are not visible for the fusion protein. The Cx36 signal (lane 5) is extremely weak and nearly not visible but becomes apparent after immunoprecipitation (IP; **B**, lane 5). **(B)** IP using the same HeLa cell transfections as in **(A)**. Single or double transfections with untagged connexins did not show any bands in IP, as expected (lanes 1, 3, 9). Most importantly, when cells expressed Cx36-EGFP and untagged Cx30.2, Cx30.2 was detected in the IP (lane 7, lowest blot, open arrowhead); similarly, when cells expressed the Cx30.2-EGFP fusion protein together with Cx36, a band appeared for the native Cx36 (lane 8, middle blot, open arrowhead). These results suggest interaction of Cx30.2 and Cx36 in HeLa cells. Please note that the anti-Cx36 antibody produced several unspecific bands. The strong band at 25 kDa **(B)** presumably originates from the light chain of the GFP antibody from the magneto-beads, reacting with the secondary antibody. The origin of the strong double band appearing in lane 8 **(B)** is unclear. For detection with anti-Cx30.2 **(A,B)**, the same blot was used as for Cx36 after stripping.

Although the vast majority (94%) of Cx30.2-expressing, parvalbumin-positive interneurons in the hippocampus also expresses Cx36 (Kreuzberg et al., 2008), interaction of Cx30.2 and Cx36 has never been shown. In contrast, Cx36 was assumed to only form homomeric connexons (Teubner et al., 2000; Li et al., 2008). Therefore, we aimed to investigate whether Cx30.2 and Cx36 are able to interact and form heteromeric gap junctions. As commercially available antibodies give the same staining in wild-type and Cx30.2 lacZ retina (Müller et al., 2010a), we generated polyclonal antibodies in rabbits, using a peptide derived from the last 20 amino acids of the C-terminal end of mouse Cx30.2. This peptide shows no sequence homology with other connexin isoforms. Specificity of the affinity-purified antibodies was evaluated on HeLa cells, transfected with different connexin constructs: Cx36 and Cx30.2, expressed either alone or in combination with Cx36-EGFP and Cx30.2-EGFP fusion proteins (Figure 4A). Western blot analysis of HeLa cell extracts revealed bands of 32–36 kDa for Cx30.2 and 58 kDa for Cx30.2-EGFP fusion proteins when blots were probed with our newly generated anti-Cx30.2 antibodies (Figure 4A, bottom). The native Cx30.2 showed several immunoreactive bands in the range of 32–36 kDa (Figure 4A, bottom, open arrowhead), slightly higher than the expected size of 30 kDa, suggesting different phosphorylation states. Treatment with alkaline phosphatase proved that these bands are indeed due to phosphorylation (Supplementary Figure 1). Probing the same blots with anti-EGFP antibodies verified the specificity of Cx30.2-immunoreactive signals (Figure 4A, lanes 4, 6). Notably, EGFP-positive bands occurred at a higher molecular weight for Cx30.2-EGFP constructs than Cx36-EGFP constructs, pointing to differences in phosphorylation states (Figure 4A, filled arrowheads, top). Cross-reactivity with Cx36 was excluded because anti-Cx30.2 antibodies did not recognize Cx36 in Cx36-transfected HeLa cell extracts (Supplementary Figure 2A).

**Figure 5:**
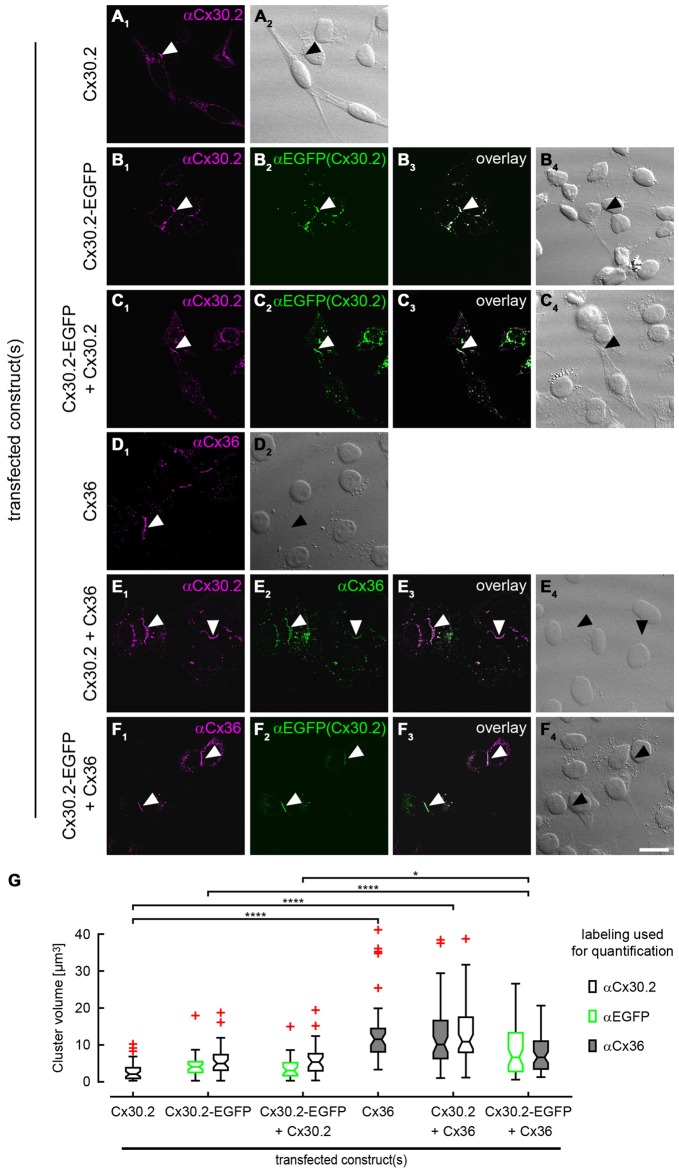
**Cx30.2 and Cx36 can assemble into the same gap junctional plaques in HeLa cells.** HeLa cells transfected with Cx30.2 **(A)**, Cx30.2-EGFP **(B)**, Cx30.2 + Cx30.2-EGFP **(C)**, Cx36 **(D)**, Cx30.2 + Cx36 **(E)** and Cx30.2-EGFP + Cx36 **(F)**. For Cx30.2 detection, our newly generated rabbit antibodies were used. Cx30.2 and Cx36 appear in the same gap junctional plaques when co-expressed **(E,F)**. Please note the size differences for the different transfections. Gap junction plaques containing only variants of Cx30.2 **(A–C)** were rather small compared to gap junction plaques containing the native Cx36 and Cx30.2 variants **(D–F)**. Controls with tagged and untagged Cx variants served to control for effects of the EGFP tag (as shown by Helbig et al., 2010 for Cx36-EGFP). Arrowheads point to gap junction plaques between two adjacent HeLa cells. **(G)** Boxplots representing the volume of gap junction clusters in the different HeLa cell transfections, determined with antibodies for Cx30.2, EGFP and Cx36. Red crosses represent outliers. Please note that the two detection channels in individual transfections were never significantly different (*p* > 0.999). *n* = 38–58 gap junction clusters from three different transfection assays. *p < 0.05; ****p < 0.0001. Scale bar: 20 μm.

Antibody specificity was also tested in immunostainings of HeLa cells (Figure 5) transfected with Cx30.2-EGFP alone. The native EGFP and antibodies directed against Cx30.2 showed the same pattern and almost complete overlap of both signals, again verifying the specificity of the Cx30.2 antibodies (Figure 5B). Untransfected cells were void of labeling (see Supplementary Figure 2A).

As rabbit anti-Cx30.2 antibodies did not work in tissue (see next paragraph), we also generated anti-Cx30.2 antibodies in guinea pig against amino acids 92–109 of the cytoplasmic loop (Table 1). Compared to the rabbit antibodies, guinea pig antibodies gave similar results in Western Blot and HeLa cell experiments (Supplementary Figure 3). Although the peptide showed 50% identity with mouse Cx36, no cross-reactivity with Cx36 was observed (Supplementary Figure 3).

### Interaction of Cx30.2 and Cx36

Unfortunately, none of our anti-Cx30.2 antibodies worked on mouse cryosections although different tissues (heart, retina, brain) were tested with different fixation and incubation protocols (4% vs. 2% PFA, PBS, PB, TBS with or without triton-X100 etc.) and different epitope unmasking techniques (heat and citrate buffer). It is possible that the immunogenic regions contain phosphorylation sites or may be complexed with other proteins. This prevented us from directly examining the co-expression and potential interaction of both connexins in AII amacrine cells and other brain areas.

However, to test whether Cx30.2 and Cx36 can principally interact, we performed co-IP experiments. HeLa cells were transfected either with Cx30.2, Cx36, Cx30.2-EGFP and Cx36-EGFP alone or, as double transfection, in all possible combinations (except for Cx30.2-EGFP with Cx36-EGFP; see Figure 4) to control for effects of the EGFP tags (Helbig et al., 2010). GFP antibodies coupled to magnetic beads were used to isolate GFP-containing connexins from HeLa cell extracts. As expected, non-tagged connexin variants (lanes 1, 3, 9) were detected with the respective anti-Cx antibodies in extracts (Figure 4A, open arrowheads) but not in immunoprecipitates (Figure 4B), whereas the single fusion proteins showed bands in extracts and immunoprecipitates (Figures 4A,B, filled arrowheads, lanes 2, 4), with the Cx36-EGFP band running slightly lower than the expected 64 kDa. Moreover, when HeLa cells were transfected with equal amounts of the tagged and untagged connexin variant (Cx30.2 + Cx30.2-EGFP and Cx36 + Cx36-EGFP, lanes 5 and 6, respectively), both proteins were detected after IP (Figure 4B)—as expected when both variants are inserted into the same gap junction channel, demonstrating the reliability of the IP protocol. Most importantly, when HeLa cells expressed Cx36-EGFP together with Cx30.2, Cx30.2 was immunoprecipitated together with the EGFP-tagged Cx36 construct (Figure 4B, lane 7). Conversely, Cx36 was pulled down via Cx30.2-EGFP when cells co-expressed both constructs (Figure 4B, lane 8).

To exclude that the interaction of Cx30.2 and Cx36 only occurred via the GFP tag, we performed IP experiments with the untagged proteins expressed in HeLa cells (Supplementary Figure 4). When Cx30.2 and Cx36 were co-expressed, Cx36 was pulled down with Cx30.2 antibodies coupled to beads (Supplementary Figure 4, asterisk). When HeLa cells were only transfected with Cx36, the pull-down was negative, as expected for this control condition. In summary, these results demonstrate that Cx36 and Cx30.2 are able to interact and presumably form heteromeric channels in HeLa cells.

To confirm these results, we analyzed the same combinations of constructs as used for co-IP with immunolabeling of HeLa cells (Figure 5), in three independent transfection experiments. In transfections with only Cx30.2 variants (Figures 5A–C, arrowheads), gap junction plaques were rather small, did not differ in size for EGFP-tagged and untagged Cx30.2 but were significantly smaller than when Cx36 was expressed alone (Figures 5D,G). When Cx30.2 variants were co-expressed with Cx36 (Figures 5E,F, arrowheads), Cx30.2-containing gap junction plaques became significantly larger (Figure 5G) when compared to Cx30.2 variants alone. This indicates that Cx30.2 is incorporated into the larger gap junction plaques made of Cx36. Together with the complete overlap of both immunosignals, this again suggests interaction of Cx36 and Cx30.2; however, other explanations cannot be excluded. The volume occupied by the immunoreactivities for Cx36 and Cx30.2 was the same when Cx36 and Cx30.2 were co-expressed (Figure 5F; *p* > 0.999), suggesting the formation of heteromeric gap junction channels. Naturally, we cannot exclude the formation of heterotypic gap junction channels with Cx36-containing hemichannels provided by one cell and Cx30.2-containing hemichannels by the other. However, immunosignals for Cx36 and Cx30.2 variants do not only overlap in the plasma membrane, at the gap junction plaque, but also in intracellular vesicles which presumably transport individual connexin hemichannels to the membrane. Thus, we hypothesize the formation of heteromeric rather than heterotypic Cx36/Cx30.2 gap junction channels.

In summary, we provide the first biochemical evidence that Cx30.2 can interact with Cx36 and presumably form heteromeric gap junctions.

### Assessing the Primary Rod Pathway in Cx30.2-Deficient Mice

**Figure 6:**
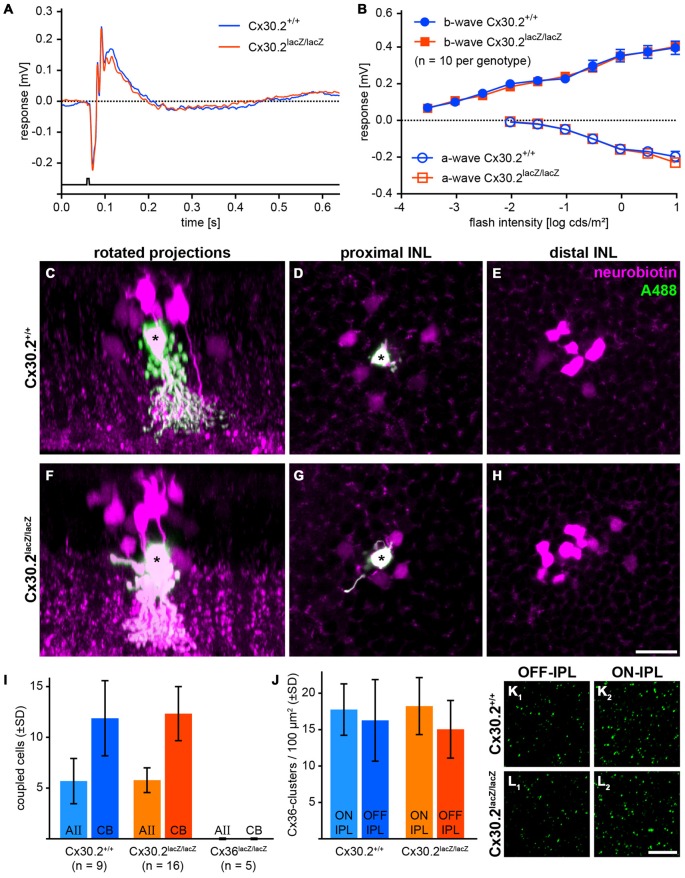
**In Cx30.2^lacZ/lacZ^ mice, the primary rod pathway is unaffected from lack of Cx30.2. (A,B)** Electroretinograms under scotopic conditions did not differ between Cx30.2^lacZ/lacZ^ mice and their wild-type littermates. The amplitudes of a- and b-wave did not show any significant differences. **(C–H)** Representative results of AII cell tracer-coupling experiments (asterisks represent Alexa-Fluor-488-injected AII cells; the tracer neurobiotin is shown in magenta). Rotated projections show the injected AII amacrine cell (asterisk) and neurobiotin-containing coupled cells **(C,F)**. Whole-mount views of an AII amacrine cell (asterisk) with its tracer-coupled partners in the proximal and distal INL, representing AII **(D,G)** and ON cone bipolar cells **(E,H)**, respectively. **(I)** The amount of tracer coupling of AII amacrine cells did not differ between Cx30.2-deficient and wild-type mice. In contrast, coupling to both AII amacrine and ON cone bipolar cells was completely abolished when Cx36 was knocked out. **(J–L)** Lack of Cx30.2 was not compensated by upregulation of Cx36 in the OFF **(K_1_,L_1_)** or ON sublamina of the IPL **(K_2_,L_2_)**. The number of Cx36-positive clusters did not differ between genotypes. Scale bars: **(C–H)** 20 μm; **(K,L)** 10 μm.

As we found Cx30.2 expression in AII amacrine cells of the mouse retina and HeLa cell experiments suggest the formation of heteromeric Cx36/Cx30.2 gap junction channels, we aimed to analyze the effects of Cx30.2-deficiency on the primary rod pathway. AII amacrine cells form two different sets of gap junctions, both of which we tested for functionality. To assess signal transmission from AII amacrine cells via heterocellular gap junctions to ON cone bipolar cells, we recorded scotopic ERGs (Figures 6A,B). If these junctions were affected by the lack of Cx30.2, the b-wave amplitude, reflecting the summed potential of depolarizing (ON) bipolar cells in response to a brief flash of light, should be reduced, as was reported for Cx36- and Cx45-deficient retinas (Güldenagel et al., 2001; Maxeiner et al., 2005). However, scotopic ERGs were unaffected: a- and b-wave amplitudes were the same for Cx30.2-expressing and -deficient mice (Figure 6B). These data suggest that AII/ON cone bipolar cell gap junctions are completely functional in Cx30.2-deficient mice.

Next, we injected the gap junction-permeant tracer neurobiotin into individual AII amacrine cells and compared tracer spread between genotypes. Again, we found no significant differences: AII amacrine cells were coupled on average to six other AII amacrine cells and 12 ON bipolar cells under our injection conditions (Figures 6C–I), whereas coupling to both cell types was completely abolished when Cx36 was knocked out (Figure 6I). These data indicate that also AII/AII gap junctions are functional in Cx30.2-deficient mice.

To test whether lack of Cx30.2 was compensated by upregulation of Cx36, we quantified Cx36 immunoreactivity in the ON and OFF sublamina of the IPL in controls and Cx30.2-deficient mice. However, again no significant differences were found between genotypes (Figures 6J–L).

Taken together, these data suggest that Cx30.2 only plays a minor role in the primary rod pathway. However, it may serve a regulatory function or represent a substrate for the observed differences between homo- and heterocellular gap junctions made by AII amacrine cells.

## Discussion

This study provides, to the best of our knowledge, the first biochemical demonstration that Cx30.2 is able to interact with Cx36 in HeLa cells. Moreover, we find Cx30.2 to be expressed in AII amacrine cells and ipRGCs in the mouse retina. Despite its expression in AII cells, the relay interneurons of the primary rod pathway, scotopic ERGs were not impaired and tracer coupling of AII cells was unaltered in Cx30.2-deficient mice. We hypothesize that Cx30.2 may rather have a regulatory function for Cx36-containing gap junctions in AII cells than being important for rod signal transmission.

### Potential Heteromerization of Cx30.2 and Cx36

In the mouse brain, the expression profiles of Cx36 and Cx30.2 are similar, with a high degree of co-expression in striatum, hippocampus and cortex (Kreuzberg et al., 2008). Both connexins are also found in β-cells of the pancreas (Coronel-Cruz et al., 2013) and chromaffin cells of the adrenal medulla (Kreuzberg et al., 2008). Despite the extensive co-distribution of the two connexins, none of the previous studies examined whether they could form heteromeric Cx30.2/Cx36 gap junction channels. In contrast, Cx36 was assumed to only form homomeric and homotypic gap junction channels (Teubner et al., 2000; Li et al., 2008) whereas Cx30.2 was shown to form heteromeric channels with other cardiac connexins (Gemel et al., 2008).

Using newly generated anti-Cx30.2 antibodies, we demonstrate that Cx30.2 can assemble with Cx36 into gap junction channels in HeLa cells. Evidence comes from three independent sets of transfection experiments in HeLa cells: (1) (co)-IP with EGFP-tagged connexins via anti-EGFP antibodies coupled to magnetobeads; (2) (co)-IP of Cx36 via anti-Cx30.2 antibodies coupled to beads; and (3) immunofluorescence quantification of gap junction plaque size. IP of Cx36-EGFP led to pull-down of Cx30.2 when both connexins were co-expressed. Conversely, Cx36 was pulled down when co-expressed with Cx30.2-EGFP. We excluded that the interaction is only indirect via the GFP tag by demonstrating pull-down of Cx36 upon co-expression with Cx30.2. Additionally, we quantified the size of gap junction plaques formed in HeLa cells transfected with Cx30.2 variants alone and in co-expression with Cx36. Gap junction plaques containing Cx30.2 (or its EGFP-tagged variant) alone were significantly smaller in volume than when Cx36 was co-expressed. Moreover, as expected for heteromeric channels, both connexins occupied the same volume when co-expressed (*p* > 0.999).

We cannot entirely exclude, however, that Cx30.2 and Cx36 form bi-homotypic gap junctions, as is debated for Cx45 and Cx36 in the retina (Li et al., 2008). In this case, a protein binding to Cx30.2 and Cx36 would co-scaffold both connexins and thus promote the formation of larger Cx30.2 homotypic gap junction plaques when Cx36 and Cx30.2 are co-expressed. However, bi-homotypic Cx30.2 and Cx36 intercellular channels would colocalize and occupy the same volume only if the affinity of the scaffolding protein for both connexins is exactly the same. Thus, the presence of bi-homotypic Cx30.2 and Cx36 gap junctions seems highly unlikely.

Together, these data provide evidence for interaction of Cx36 and Cx30.2 in HeLa cells and suggest the formation of heteromeric channels.

### Cx30.2-Expressing Cell Types

In the mammalian retina, expression of Cx30.2 was shown in six different types of ganglion cells and so far undefined groups of amacrine cells (Müller et al., 2010a). Here, we further characterized Cx30.2-expressing neurons in the mouse retina, using *lacZ* expression in Cx30.2^*lacZ*^ mice as a proxy. This is a feasible approach because earlier studies from Kreuzberg et al. (2006) and Müller et al. (2010a) also used *lacZ* labeling to identify Cx30.2-expressing cells and confirmed Cx30.2 expression functionally as a slowing of conductance velocity in the heart and loss of tracer coupling in retinal ganglion cells in Cx30.2-deficient mice, respectively. As we could not directly reveal the Cx30.2 protein, we confirmed Cx30.2 expression by RT-PCR from wild-type retinas (Figure 1A).

*LacZ*-expressing cells were numerous in the proximal INL and the GCL. They showed a high degree of co-expression with the calcium-binding proteins parvalbumin and calbindin in both layers and calretinin in the GCL, consistent with Cx30.2 expression in multiple types of amacrine and ganglion cells. Pang et al. (2013) recently demonstrated that heterotypic amacrine/ganglion cell coupling is not completely abolished in Cx36/Cx45-deficient mice. Therefore, it seems likely that Cx30.2 electrically couples amacrine and ganglion cells, which we also demonstrated earlier for RGA1 ganglion cells (Müller et al., 2010a). However, the functional role of the coupling between diverse types of amacrine and ganglion cells is still largely unknown (see also below, Pang et al., 2013).

### Cx30.2 is Expressed in Intrinsically Photosensitive Ganglion Cells (ipRGCs)

ipRGCs express the photopigment melanopsin and were shown to mediate non-image-forming visual functions, such as pupillary constriction and circadian photoentrainment (Güler et al., 2008). The mouse retina contains five different types of ipRGCs (M1–5; Sand et al., 2012), with types M1–3 immunoreactive to melanopsin. Calcium imaging showed that ipRGCs form networks that could be uncoupled by the gap junction blocker carbenoxolone (Sekaran et al., 2003). The presence of gap junctions on ipRGCs was later verified by tracer injections: types M1–3 show tracer coupling to GABAergic, displaced wide-field amacrine cells (Müller et al., 2010b) and Cx30.2 was suggested as the underlying connexin. Our data support this suggestion because double labeling for β-gal and melanopsin demonstrated a high degree of co-expression in ganglion cells in the INL and GCL. As most ganglion cells are coupled via Cx36 (Pan et al., 2010), it seems possible that Cx36 and Cx30.2 are both expressed in some ipRGCs. However, M2 ganglion cells were proposed to lack Cx36 (Pan et al., 2010) and correspond to RGA1 cells (Sun et al., 2002) which were earlier described to be coupled to two different types of polyaxonal amacrine cells (Völgyi et al., 2009). Using tracer injections, we showed before Müller et al. (2010a) that RGA1 (M2) cells lose their coupling when Cx30.2 is missing, suggesting that M2 cells form gap junctions containing only Cx30.2 on the M2 cell side. M3 cells which were also shown to be coupled to displaced amacrine cells are bistratified neurons (Müller et al., 2010b). As these ipRGCs are also immunoreactive to melanopsin it is likely that they also express Cx30.2 because of the strong co-expression of Cx30.2 and melanopsin. Interestingly, Cx45 was found to be expressed in bistratified ganglion cells (Schubert et al., 2005) raising the possibility that Cx30.2 and Cx45 are co-expressed. As Cx45 was shown to be able to heteromerize with Cx30.2 (Gemel et al., 2008), interaction with Cx45 could also be a potential function of Cx30.2 in ipRGCs.

Coupling between amacrine and ganglion cells was generally considered to enlarge receptive fields and synchronize firing of ganglion cells. However, Reifler et al. (2015) recently showed that ipRGCs do not receive input from but instead provide input to displaced amacrine cells via gap junctions. Thus, Cx30.2 may serve to propagate the sustained light responses from ipRGCs to displaced, wide-field amacrine cells, potentially enabling ipRGCs to regulate the secretion of neuromodulators in displaced amacrine cells (Reifler et al., 2015).

### Cx30.2 is Expressed in AII Amacrine Cells of the Mouse Retina

Among the Cx30.2-expressing neurons in the INL, we found AII amacrine cells. Dye-injecting pre-labeled β-gal-positive neurons revealed the distinctive AII morphology with lobular appendages in the OFF- and arboreal dendrites in the ON-sublamina of the IPL. Disabled-1 staining, labeling only AII cells in the mouse retina (Rice and Curran, 2000), confirmed *lacZ* expression in the relay neurons of the primary rod pathway. Also, the high density of β-gal-positive neurons in the proximal INL (3500 cells/mm^2^, averaged over different eccentricities) is consistent with expression in AII amacrines, which represent the most numerous amacrine cell in the mouse retina (~2600 cells/mm^2^, Rice and Curran, 2000). We also found *lacZ* expression in amacrine cells whose somata were more distal than AII cell somata or between AII somata, suggesting—together with the density difference between *lacZ*-positive cells and AII cells—the presence of at least one other Cx30.2-expressing amacrine cell type in the mouse retina.

### A Potential Regulatory Role for Cx30.2 in AII Amacrine Cells?

We found Cx30.2 expression in AII amacrine cells and demonstrated in HeLa cells that Cx30.2 and Cx36 are likely able to assemble into the same gap junction channel. Therefore, we analyzed whether the primary rod pathway was affected in Cx30.2-deficient mice. However, ERG recordings and tracer injections revealed no changes in b-wave amplitudes or the number of cells coupled to the injected AII cell, respectively. These data suggest that AII/AII amacrine and AII/ON bipolar cell coupling are fully functional in Cx30.2-deficient mice. Results are similar to data obtained from hippocampal neurons which revealed—despite strong expression of Cx30.2—no phenotype when Cx30.2 was missing (Kreuzberg et al., 2008). As hippocampal neurons and AII amacrine cells also express Cx36, lack of Cx30.2 may be compensated by Cx36. Therefore, we tested for upregulation of Cx36 in Cx30.2-deficient mice but did not find an increase in Cx36 immunoreactivity in the IPL.

We also confirmed earlier results (Deans et al., 2002; Meyer et al., 2014) that lack of Cx36 completely abolished coupling in AII amacrine cells. As HeLa cell transfections revealed significantly smaller gap junction plaques for Cx30.2 than for Cx36, Cx36-deficient AII cells may not form enough Cx30.2 gap junction channels to allow for tracer to spread from one cell to another. This is supported by ERG recordings which showed dramatically reduced b-wave amplitudes in Cx36-deficient mice, suggesting that residual electrical coupling between AII cells and ON bipolar cells by Cx30.2 homomers is rather unlikely. Yet, as we cannot determine the subcellular localization of Cx30.2, we cannot completely exclude that Cx30.2 forms homomeric channels in AII cells. This could be the case if Cx30.2 and Cx36 or Cx45 were transported to different compartments or different gap junctional plaques within the AII cell or assembled into bihomotypic gap junction plaques as suggested for Cx36 and Cx45 (Li et al., 2008).

Cx36 and Cx30.2 exhibit a low sensitivity to changes in transjunctional voltage and have a rather low unitary conductance (Cx30.2: 9 pS; Cx36: 14 pS; Teubner et al., 2000; Kreuzberg et al., 2005), suggesting that they have quite similar properties. However, both connexins differ in the kinases they are phosphorylated by: in the retina, Cx30.2 was shown to be modulated by protein kinase C (PKC; Müller et al., 2010a) and also contains a putative protein kinase G (PKG) phosphorylation site. In contrast, Cx36 is phosphorylated by CamKII (Kothmann et al., 2012). Similarly, the two sets of gap junctions formed by AII cells (AII/AII and AII/ON bipolar cell gap junctions) are differentially regulated (Mills and Massey, 1995). Thus, it is tempting to speculate that Cx30.2 may provide a substrate for the differential regulation of gap junctions in AII amacrine cells, rendering AII gap junctions (or maybe even only one of its two sets) sensitive to PKC- or PKG-mediated regulation. If this is indeed the function of Cx30.2, why do we not see altered dye coupling? Phosphorylation of gap junctions is not a global process in AII cells but occurs rather locally, differentially affecting individual gap junction plaques as shown by Kothmann et al. (2009). Thus, we believe that evaluating global tracer coupling is not sensitive enough to detect the minor differences in gap junction conductance that are to be expected by changes in phosphorylation. Moreover, AII amacrine cells are coupled to most if not all types of ON bipolar cells (Veruki and Hartveit, 2002), which differ in their physiology. It seems therefore conceivable that gap junctional signal transmission from AII cells is not the same for all ON bipolar cell types but slightly differs between types, for example by connexin composition or phosphorylation state.

Cx30.2 is widespread in neurons in the inner retina and can interact with Cx36 in HeLa cells. In AII amacrine cells, we suggest that Cx30.2 may confer sensitivity to PKC-mediated pathways to the electrical synapse and thus provide a means for dynamic regulation of electrical signal transmission between rod and cone pathways.

## Author Contributions

KD conceived the project; KD, UJ-B, AM, SGH and RW designed experiments; AM, HG, ST, JS, BD, KD and SGH performed experiments; all contributed to the interpretation of data; KD wrote the article with help from AM; AM, KD and ST prepared the figures; all authors edited and commented on the manuscript.

## Funding

This work was funded by a grant from the Deutsche Forschungsgemeinschaft to KD (DE1154/5-1) and UJ-B (JA 854/3-1). AM received a stipend from the PhD programme Neurosenses (Ministry for Science and Culture of Lower Saxony, Germany). JS received a stipend from the Deutsche Forschungsgemeinschaft (Research Training Group 1885/1).

## Conflict of Interest Statement

The authors declare that the research was conducted in the absence of any commercial or financial relationships that could be construed as a potential conflict of interest.

## Abbreviations

Cx: connexin
GCL: ganglion cell layer
INL: inner nuclear layer
IPL: inner plexiform layer
ipRGC: intrinsically photosensitive retinal ganglion cell
PB: phosphate buffer
PFA: paraformaldehyde.
