# Supplementary material for: Connexin30.2: In Vitro Interaction with Connexin36 in HeLa Cells and Expression in AII Amacrine Cells and Intrinsically Photosensitive Ganglion Cells in the Mouse Retina
Source: Front Mol Neurosci. 2016 May 27;9:36. doi: 10.3389/fnmol.2016.00036 (PMC4882342; doi:10.3389/fnmol.2016.00036)
Supplement: Supplementary file 1 [file Presentation_1.pdf]

Supplementary Material

Connexin30.2: *in vitro* interaction with connexin36 in HeLa cells and expression in AII amacrine cells and intrinsically photosensitive ganglion cells in the mouse retina

Arndt Meyer[1,4], Stephan Tetenborg[1], Helena Greb[1], Jasmin Segelken[1], Birthe Dorgau[1,5], Reto Weiler[1,3], Sheriar G. Hormuzdi[2], Ulrike Janssen-Bienhold[1,3], Karin Dedek[1,3,4]*

*** Correspondence:** Corresponding Authors: Karin Dedek, karin.dedek@uni-oldenburg.de, Ulrike Janssen-Bienhold, ulrike.janssen.bienhold@uni-oldenburg.de, Sheriar G. Hormuzdi, s.g.hormuzdi@dundee.ac.uk

1 Supplementary Figures

1.1 Supplementary Figures

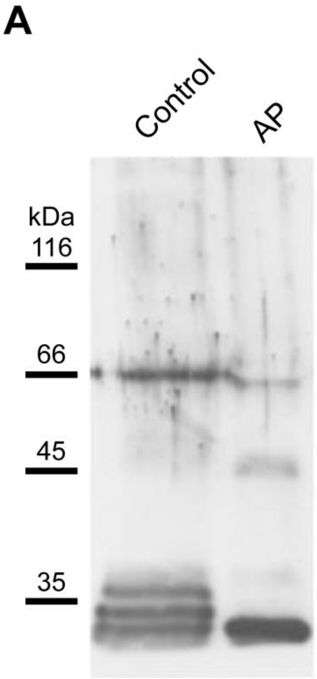

Supplementary Figure 1. Multiple Cx30.2 bands represent phosphorylated Cx30.2 isoforms. Western blot of total homogenates from Cx30.2-transfected HeLa cells, probed with our anti-Cx30.2 antibodies from rabbit. Multiple Cx30.2-immunoreactive bands become visible in a range of 32-36 kDa under control conditions (control). These bands are slightly higher than the expected size of 30 kDa. Treatment with alkaline phosphatase (AP) reduced the number of Cx30.2-immunoreactive bands and enhanced the lowest band (~32 kDa), indicating that multiple bands present in control conditions represent phosphorylated Cx30.2 isoforms. This is slightly higher than the previously reported molecular weight of Cx30.2 (Nielsen and Kumar, 2003; Gemel et al., 2008).

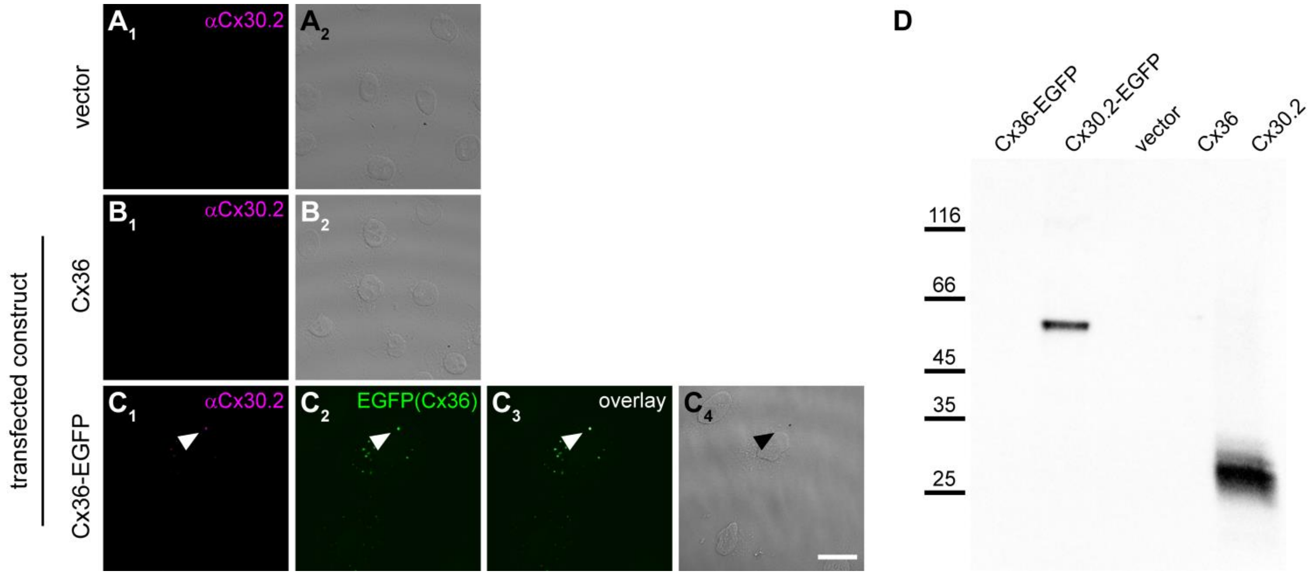

Supplementary Figure 2. Newly generated antibodies against Cx30.2 do not show cross-reactivity with Cx36 or Cx36-EGFP. HeLa cells transfected with the empty vector **(A)**, Cx36 **(B)**, and Cx36-EGFP **(C)** showed no cross-reactivity with newly generated anti-Cx30.2 antibodies from rabbit. Only very weak signals were detected in Cx36-EGFP transfectants (C, arrowhead). **(D)** Anti-Cx30.2 antibodies generated in rabbit do not cross-react with Cx36. Western blot of total homogenates from HeLa cells transfected with Cx36-EGFP, Cx30.2-EGFP, the empty vector, Cx36 and Cx30.2. The antibodies only detect the Cx30.2-EGFP fusion protein at its expected size of ~58 kDa and multiple bands around 32 kDa. Scale bar: 20 µm.

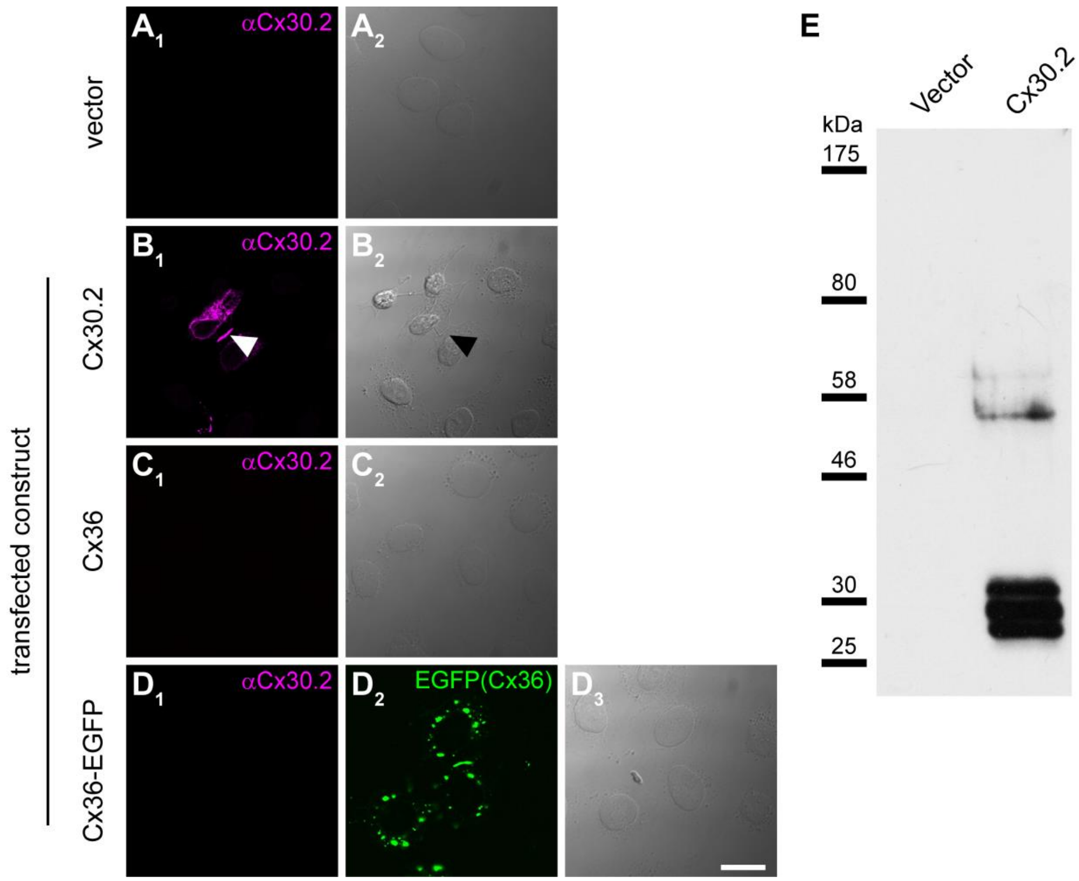

Supplementary Figure 3. Newly generated antibodies against mouse Cx30.2 raised in guinea pigs showed similar results as the antibodies raised in rabbits. (A) HeLa cells transfected with the empty vector **(A)**, Cx30.2 **(B),** Cx36 **(C)**, and Cx36-EGFP **(D)**. Anti-Cx30.2 antibodies from guinea pig only show immunoreactivity in Cx30.2-transfected cells and label gap junctions (arrowhead in B). Antibodies do not cross-react with Cx36 as Cx36-transfected cells are void of label (C, D). **(E)** Western blot of total homogenates from N2A cells transfected either with the empty vector or Cx30.2, probed with our anti-Cx30.2 antibodies from guinea pig. Multiple Cx30.2-immunoreactive bands become visible at a range of 32-36 kDa in Cx30.2-transfected cells which are absent in the control.

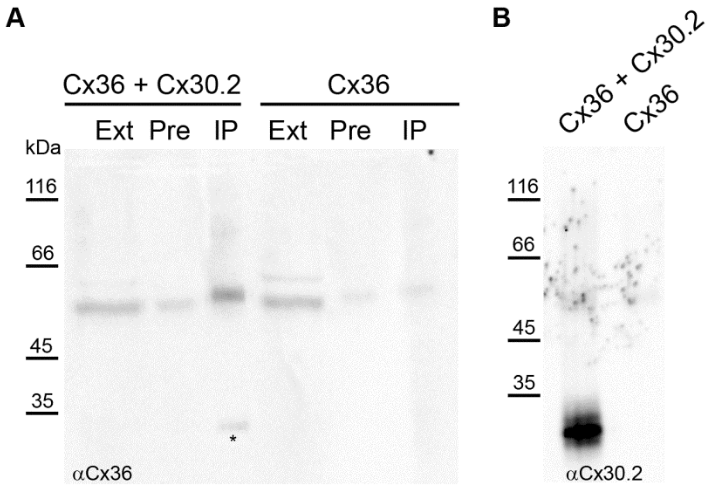

Supplementary Figure 4. Direct interaction of Cx30.2 and Cx36, revealed by co-immunoprecipitation of Cx36 and Cx30.2 using the anti-Cx30.2 antibody generated in rabbit. (A) Western Blot with pre-cleared extracts (Ext), pre-adsorbed proteins (Pre) and immunoprecipitated extracts (IP) from HeLa cells transfected with Cx36+Cx30.2 or Cx36 alone. Cx36 (asterisk) was co-precipitated when co-transfected with Cx30.2, demonstrating direct interaction**.** This band was absent in controls (Cx36 transfectants). Samples preadsorbed with protein A beads before immunoprecipitation only showed unspecific bands around 55 kDa, which probably result from protein A enrichment. Please note that the Cx36-immunoreactive band was absent from extracts presumably because of the low amounts of protein present. **(B)** IP was confirmed by immunodetection of Cx30.2 in lane Cx36 + Cx30.2 (lane 1). Anti-Cx30.2 antibodies from rabbit revealed a strong immunoreactive band (arrow). No signal was detected when only Cx36 was expressed (lane2).

2 Supplementary Methods

Immunoprecipitation with Cx30.2 antibodies and Western Blot analysis

2 µg of our newly generated anti-Cx30.2 antibodies (rabbit) were added to 50 µl of protein A microbeads and incubated with 500 µl of 0.2 M triethanolamine (pH 8) at room temperature for 10 min. Then, 55 µl of a 0.2 M stock solution of the cross-linking reagent dimethylpimelimidate *2 HCl (Thermo Fisher) were added to the mixture and incubated for 30 min. Cross-linking was stopped by adding 500 µl of 50 mM Tris (pH 8.8). This mixture was added to µMACS column (Miltenyi Biotec GmbH, Germany) and washed with PBS. Afterwards, 20 µl of heated (65 °C) IP buffer were added to the column and incubated for 2 min. Elution was done with 150 µl of IP buffer. IP buffer contained 0.5% NP-40, 20 mM Tris, 60 mM NaCl (pH 7.4), and phosphatase and protease inhibitors (Roche Diagnostics, Mannheim, Germany). After centrifugation of solubilized membrane proteins from transfected HeLa cells, the supernatant was incubated with 20 µl of protein A magnetobeads (Miltenyi) for 30 min on ice to get rid of non-specifically binding proteins. This sample was added to a µMACs column and the flow through was collected as pre-cleared extracts. 30 µl were taken as a sample for SDS-PAGE. Preadsorbed proteins were eluted from the column. Afterwards the precleared extract was incubated with 65 µl of freshly prepared anti-Cx30.2 beads for 2 h on ice.

To test whether the multiple bands visible for Cx30.2 represent phosphorylated Cx30.2 isoforms, we used alkaline phosphatase to induce dephosphorylation. HeLa cell samples (3 µg protein of total homogenates) were incubated for 1 min in a microwave to block any enzymatic activity. Afterwards, 6 µl of alkaline phosphatase (Boehringer, Mannheim, Germany) were added and the samples were incubated for ~19 hours at 37 °C and subsequently analyzed by Western blot as described (Supplementary Fig. 1). To improve the separation of the putative phosphorylated bands, run duration for this gel was prolonged for 15 min, after the dye front (bromophenol blue) had reached the bottom of the gel (after ~90 min).

3 Supplementary References

Gemel, J., Lin, X., Collins, R., Veenstra, R. D., and Beyer, E. C. (2008). Cx30. 2 can form heteromeric gap junction channels with other cardiac connexins. *Biochem. Biophys. Res. Commun.* 369, 388–394.

Nielsen, P. A., and Kumar, N. M. (2003). Differences in expression patterns between mouse connexin-30.2 (Cx30.2) and its putative human orthologue, connexin-31.9. *FEBS Lett.* 540, 151–156. doi:10.1016/S0014-5793(03)00252-7.
